# Tumour necrosis factor-alpha and interleukin-8 inhibit neutrophil migration in vitro and in vivo

**DOI:** 10.1155/S0962935192000607

**Published:** 1992

**Authors:** F. Q. Cunha, W. M. S. Cunha Tamashiro

**Affiliations:** 1 Department of Pharmacology Faculty of Medicine of Ribeirao Preto USP Ribeirao Preto SP 14049 Brazil; 2 Department of Microbiology and Immunology Institute of Biology UNICAMP Campinas SP 13081-970 Brazil

## Abstract

Pretreatment of human neutrophils with recombinant tumour necrosis factor-alpha (rTNF-α) and/or interleukin-8 (rIL-8), but not with either transforming growth factor-beta, interleukin-6 or interferon-gamma, rendered these cells less responsive to FMLP, in microchemotaxis assays. This inhibitory effect was dose dependent and more powerful when neutrophils were pretreated with a mixture of both cytokines. Intravenous injection of human rIL-8 (hrIL-8) and/or murine rTNF-α (mrTNF-α) also significantly reduced in vivo neutrophil migration into peritoneal cavities of rats stimulated with carrageenan. These data suggest that the defect in neutrophil migration during septicaemia or endotoxaemia may be the result of the continuous release of IL-8 and TNF-α into the circulation. Thus, either the selective control or blockade of releasing of these cytokines as well as of its effects on neutrophils may be clinically useful in reestablishing the cell defence mechanisms.


Mediators of Inflammation 1, 397–401 (1992)

### Tumour necrosis factor-alpha and interleukin-8 inhibit neutrophil migration *in vitro* and *in vivo*

F. Q. Cunha[1] and

W. M. S. Cunha Tamashiro[2,CA]

[1] Department of Pharmacology, Faculty of Medicine of Ribeirao Preto, USP, Ribeirao Preto, 14049, SP, Brazil; [2] Department of Microbiology and Immunology, Institute of Biology, UNICAMP, 13081-970 Campinas, SP, Brazil

[CA] Corresponding Author

**PRETREATMENT of human neutrophils with recombinant tumour necrosis factor-alpha (rTNF-α) and/or interleukin-8 (rIL-8), but not with either transforming growth factor-beta, interleukin-6 or interferon-gamma, rendered these cells less responsive to FMLP, in microchemotaxis assays. This inhibitory effect was dose dependent and more powerful when neutrophils were pretreated with a mixture of both cytokines. Intravenous injection of human rIL-8 (hrIL-8) and/or murine rTNF-α (mrTNF-α) also significantly reduced *in vivo* neutrophil migration into peritoneal cavities of rats stimulated with carrageenan. These data suggest that the defect in neutrophil migration during septicaemia or endotoxaemia may be the result of the continuous release of IL-8 and TNF-α into the circulation. Thus, either the selective control or blockade of releasing of these cytokines as well as of its effects on neutrophils may be clinically useful in reestablishing the cell defence mechanisms.**

**Key words:** Human neutrophils, Interleukin-8, Tumour necrosis factor-α

### Introduction

Several studies have established the fact that Gram-negative bacteraemia or circulating endotoxin decreases the ability of neutrophils (PMN) to migrate to inflammation sites.[1–3] This effect may play an important role in the evolution of septicaemia.[1,4] Neutrophil inhibitory factors have been found in many other illnesses, such as AIDS,[5] Hodgkin's disease,[6] diabetes mellitus[7] and lepromatous leprosy.[8] The source of these factors has not yet been satisfactorily demonstrated. It has been shown that macrophages incubated with *Staphylococcus aureus* release a factor(s) which inhibits neutrophil migration *in vitro*.[9,10] Recently it has been shown that the supernatant of rat macrophages pretreated with lipopolysaccharide (LPS) administered intravenously (i.v.) suppresses the recruitment of neutrophils to the peritoneal exudate induced by various inflammatory stimuli, thus mimicking the effects of LPS.[11] The factor(s) present in this supernatant was named NRIF (neutrophil recruitment inhibitory factor).[11]

Tumour necrosis factor-alpha (TNF-α) and interleukin 8 (IL-8) exert a wide spectrum of activities in inflammatory reaction. They are potent stimulators of several neutrophil functions, including chemotaxis, respiratory burst, degranulation and aggregation.[12–19] Besides these pro-inflammatory effects, it was recently demonstrated that both cytokines have anti-inflammatory properties.[13,20–24] TNF-α blocks *in vitro* chemotaxis of human and rabbit neutrophils induced by several stimuli, such as f-methionyl, l-leucyl phenylalanine (FMLP) C5a and $LTB_4$[20–22,24] and IL-8 inhibits neutrophil adhesion to cytokine activated endothelial monolayers resulting in the protection of these cells from neutrophil mediated damage *in vitro*.[23] It was also shown that the intravenous injection of TNF inhibited C5a induced neutrophil emigration into skin of mice,[24] and that systemic administration of IL-8 caused a similar effect in the skin of rabbits challenged with FMLP, IL-1, C5a or $LTB_4$.[25]

In the present investigation the effect of TNF-α and IL-8, alone or in combination, on neutrophil migration has been investigated *in vitro*. The effect of transforming growth factor-β, interleukin-1β, interleukin-6 and interferon-gamma were also tested. Since TNF-α and IL-8 inhibited neutrophil chemotaxis induced by FMLP, the effect of intravenous administration of these cytokines on carrageenan induced neutrophil recruitment in peritoneal cavities of rats was also tested.

### Materials and Methods

*Animals:* Adult male Sprague-Dawley rats weighing 150–180 g were obtained from Charles River Breeding Laboratories (Wilmington, MA).

*Reagents:* The following human recombinant cytokines were used: tumour necrosis factor-alpha (hrTNF-α) and interleukin-8 (hrIL-8) from Genentech Inc. Transforming growth factor-beta (hrTGF-β), interleukin-1beta (hrIL-1β), interferon-gamma (hrIFN-γ) and interleukin-6 (hrIL-6) from

© 1992 Rapid Communications of Oxford Ltd

Genzyme Corp. (Boston, MA). Murine recombinant tumour necrosis factor-alpha (mrTNF-α) from Genentech Inc. was used in some experiments.

In vitro *neutrophil migration:* Preparation of human neutrophils: Viable human neutrophils were obtained from heparinized venous blood of healthy subjects by monopolyresolving medium (Flow Laboratories) fractionation. The isolated neutrophils were washed three times in RPMI 1640 medium and then suspended in the same medium containing 0.1% bovine serum albumin (BSA; Sigma, St Louis).

Pretreatment of neutrophils: Neutrophil suspensions ($10^6$ cells/ml) were incubated for 30 min, at 37°C in a humidified incubator with 5% $CO_2$, either in the absence or in the presence of various concentrations of human recombinant cytokines ($10^{-7}$–$10^{-11}$ M) (hrTNF-α, hrIL-8, hrhTGF-β, hrIL-1β, hrIFN-γ and hrIL-6), prior to testing for chemotactic response to f-methionyl,l-leucyl phenylalanine (FMLP) (Sigma, St Louis) in microchemotaxis assays. The cytokines were present throughout the assay. In some experiments, neutrophils were exposed to $10^{-7}$ M of mrTNF-α and/or hrIL-8 for 30 min and subsequently washed with RPMI medium, prior to testing for responsiveness to FMLP.

Chemotaxis assay: Chemotaxis was assessed in a 48-well microchemotaxis chambers (Neuro Probe, Cabin John, MD) separated by 5 μm pore size polyvinylpyrrolidone-free polycarbonate membranes. Twenty-five μl of FMLP ($10^{-8}$ M) diluted in RPMI containing 0.1% BSA were placed in the bottom chamber and 50 μl of PMN suspensions ($10^6$ cells/ml) were added to the top chamber. The chambers were incubated for 1 h at 37°C in a humidified incubator with 5% of $CO_2$. Subsequently, the filters were removed, fixed, and stained with Diff-Quick Stain Kit (American Scientific Products, McGraw Park, IL). Neutrophils, which migrated to the lower side of the filter were counted by using a 100 × objective in five random fields. Experiments were performed in triplicate for each variable and the mean determined. The results were expressed as number of neutrophils/field. In each experiment untreated neutrophils migrating toward FMLP were used as a positive reference.

In vivo *neutrophil migration:*

Effect of cytokines on *in vivo* neutrophil migration: Murine recombinant TNF-α and/or human recombinant IL-8 (2 μg/0.5 ml/animal) were injected i.v. into a tail vein of male rats. Thirty minutes later, the animals received an intraperitoneal (i.p.) injection of carrageenan (1 mg/3 ml of PBS; Sigma). The number of peritoneal neutrophils was evaluated 4 h after the i.p. injection. Rats were sacrificed with $CO_2$, their peritoneal cavities were washed with 10 ml of PBS containing 5 IU/ml of heparin and the total and differential cell counts were determined. The results were reported as the average number of neutrophils per cavity.

*Statistics:* The results are reported as the mean ± the standard error of the mean (S.E.M.) Values of $p$ were determined using Student's $t$-test.

### Results

Human recombinant TNF-α ($10^{-7}$ M) suppressed profoundly human neutrophil chemotaxis to FMLP. Interleukin-8, at the same concentration, was less effective at inhibiting neutrophil chemotaxis. In contrast, hrTGF-β, hrIL-1β, hrIFN-γ and hrIL-6 were ineffective (Fig. 1). The inhibitory effects of hrIL-8 and hrTNF-α on neutrophil chemotaxis were dose dependent. While hrIL-8 had significant inhibitory effect on chemotaxis only at the doses of $10^{-7}$ and $10^{-8}$ M, hrTNF-α exhibited a significant inhibitory effect upon neutrophil migration at a concentration as low as $10^{-10}$ M. The combination of hrTNF-α plus hrIL-8 had a strong inhibitory effect on neutrophil chemotaxis at all concentrations tested ($10^{-7}$ to $10^{-11}$ M) (Fig. 2).

Exposure of neutrophils to $10^{-7}$ M hrIL-8 and mrTNF-α either alone or in combination, followed by cell washing, also resulted in inhibition of neutrophil chemotaxis (Fig. 3).

Intravenous administration of mrTNF-α, hrIL-8 or a mixture of both reduced neutrophil migration induced by an i.p. injection of carrageenin. Similar to the *in vitro* results, mrTNF-α was more potent than hrIL-8 in relation to the inhibition of neutrophil migration into the peritoneal cavities of rats (Fig. 4).

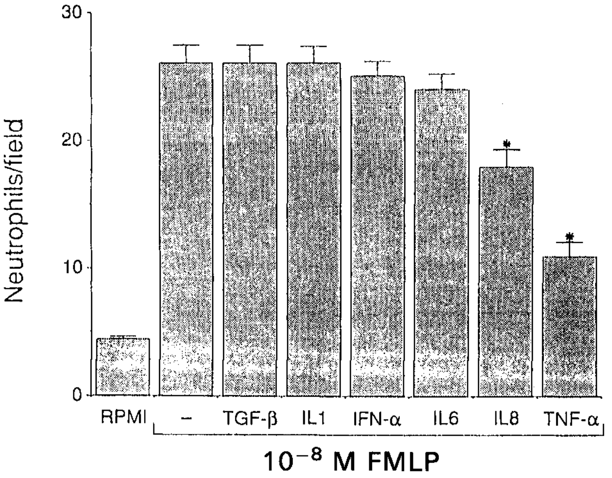

FIG. 1. Effects of cytokines on neutrophil chemotaxis induced by FMLP. The concentration of the cytokines was $10^{-7}$ M. Results are reported as means ± S.E.M. of the number of neutrophils per field. Average number of fields was 15 for each variable. (*$p < 0.01$, Student's $t$-test.)

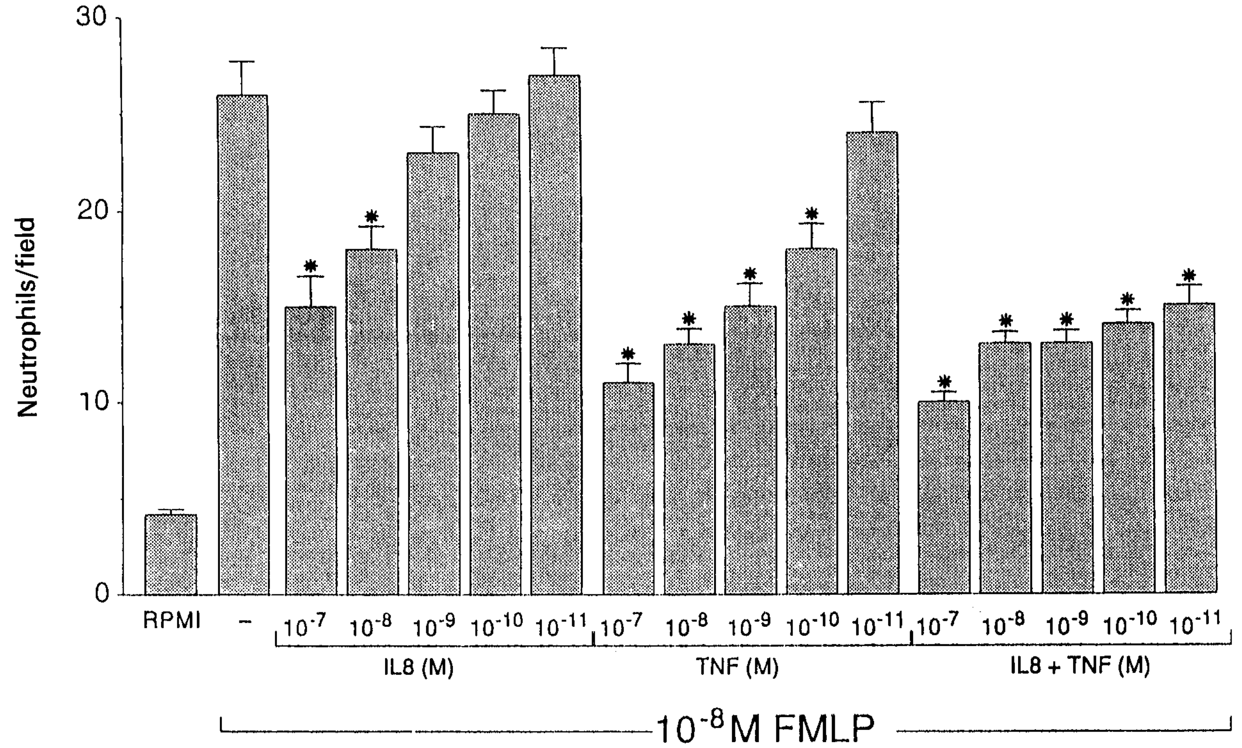

FIG. 2. Dose response effect of hrTNF-α and hrIL-8, individually or combined, on neutrophil chemotaxis induced by FMLP. Subtitles indicate molar concentration of each cytokine used either alone or in combination. Results are reported as means ± S.E.M. of the number of neutrophils per field, which were 15 on average. (*$p < 0.01$, Student's *t*-test.)

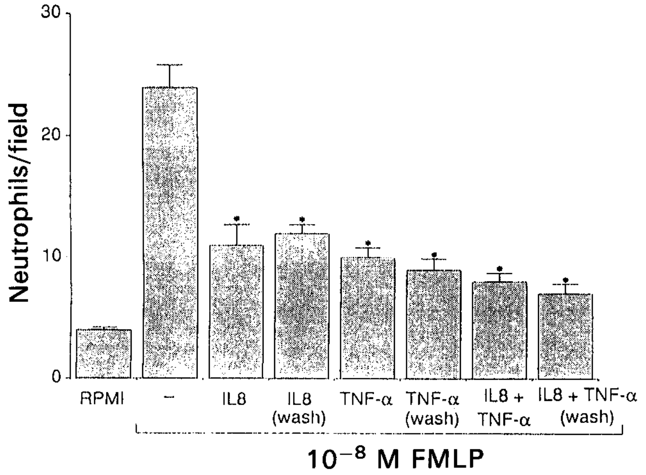

FIG. 3. Effect of pretreatment of neutrophils with hrTNF-α and hrIL-8 on neutrophil migration induced by FMLP. Cytokines were used at final concentration of $10^{-7}$ M and washed out prior to the use in microchemotaxis assay. Results are reported as means ± S.E.M. of the number of neutrophils per field. (*$p < 0.01$, Student's *t*-test.)

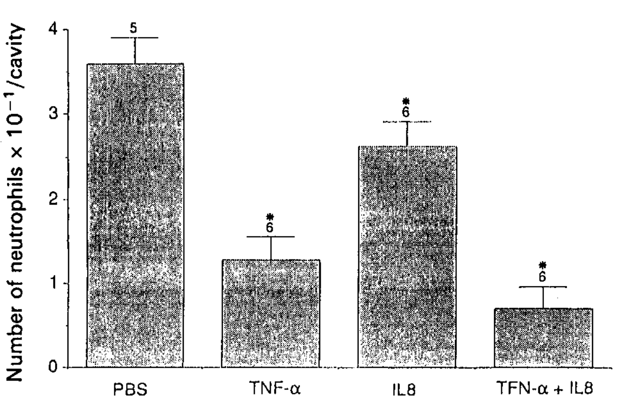

FIG. 4. Effect of intravenous pretreatment of rats with either mrTNF-α, hrIL-8 or both, upon carrageenan induced neutrophil migration into peritoneal cavities. The dose of each cytokine was 2 μg/animal. Results are means ± S.E.M. of the number of animals given above each bar. (*$p < 0.01$, Student's *t*-test).

### Discussion

Because it has been reported that both TNF and IL-8 have inhibitory effects on neutrophil functions, the effects of IL-8 and TNF-α on neutrophil chemotaxis induced by FMLP and peritoneal emigration of neutrophils induced by carrageenan were investigated. There have been controversial findings on effect of TNF-α on neutrophil migration, depending on the assay method used. TNF inhibition of neutrophil migration has consistently been seen with the agarose method,[21,26–28] while the chemotaxis chamber methods have yielded contradictory conclusions.[12,20,22,29] In the present experiments, hrTNF-α and hrIL-8 inhibited neutrophil migration *in vitro* in a dose dependent manner. While TNF-α was more potent than IL-8, the combination of these cytokines had an additive inhibitory effect. The high doses of hrIL-8 necessary to inhibit human PMN chemotaxis suggests that the cytokine is acting by crossreacting with a receptor for another ligand, or through cross-desensitization.

It is known that a high concentration of a chemotactic factor placed in the upper compartment of a chemotactic chember can inhibit neutrophil chemotaxis.[30] However, the inhibitory effect described in this paper was not due to the presence of residual TNF-α and IL-8 in the top chamber, since exposure of the neutrophils to mrTNF-α, hrIL-8 or both, followed by cell washing, had the same *in vitro* inhibitory effect.

Intravenous injection of a single dose (2 μg/animal) of hrIL-8 and/or mrTNF-α before the intraperitoneal administration of carrageenan caused significant reduction of neutrophil accumulation on rat peritoneal cavities. It has recently been reported that systemic administration of TNF[24,31] or IL-8[25,32,33] induces only a transient neutropenia which is followed by neutrophilia. Thus, the inhibitory effects observed in the present investigation probably are not due to neutropenia.

It has been shown that IL-8 blocks neutrophil adhesion to activated endothelial monolayers[23] as well as inducing the loss of lectin adhesion molecule I (LECAM-1) from the unstimulated neutrophil surface, thereby reducing neutrophil adhesion to vascular endothelium under conditions of flow.[34] These findings may explain the IL-8 induced inhibitory effect observed here. The low efficiency of IL-8 in comparison to TNF-α in rat studies perhaps may be due to a weak recognition of hrIL-8 by rat PMN. In this regard, recent studies have shown that IL-8 may have some species specificity and then its chemotactic potency can vary according to the PMN source.[35]

Previous studies have suggested that TNF-α inhibits neutrophil migration *in vitro* by increasing the expression of the CD11b, thus resulting in enhancement of neutrophil adhesion and suppression of migration.[20] The authors showed that the monoclonal antibody 60.1, which is directed against an epitope on the alpha chain of the CD11b/CD18 complex,[36] completely blocked the inhibition of neutrophil migration induced by hrTNF-α. This monoclonal antibody also completely reversed the hrTNF-α induced hyperadherence to gelatin-coated wells.[20] The TNF-α induced inhibition of neutrophil emigration into peritoneal cavities described in this paper, may also be due to the hyperadherence of neutrophils in the circulation which impairs the migration of these cells into extravascular space.

TNF-α has been implicated as an important mediator of Gram-negative sepsis, which involves extensive polymorphonuclear mediated vascular and tissue damage.[37] TNF-α injection into experimental animal produces a virtually identical 'septic shock syndrome' to endotoxin administration.[38] The animals passively immunized against TNF-α survive an otherwise lethal dose of endotoxin.[39] It has recently been shown that IL-8 also appears in the circulation of primates during septic shock, sublethal endotoxaemia and after IL-1 administration.[40] Thus, several events observed in the endotoxaemia, such as impairment of neutrophil migration, may be induced by release of IL-8 and TNF-α. Recently Cunha and coworkers,[11] have suggested that failure of neutrophils to migrate during septicaemia or endotoxaemia may be the result of a continuous release of a recruitment inhibitory factor (NRIF). Since NRIF is present in the supernatant of LPS pretreated macrophages, and since LPS also stimulates macrophages release IL-8 and TNF, probably part of inhibitory effect of the macrophage crude supernatants is due to the presence of TNF-α and IL-8 in those samples.

In conclusion, the present findings suggest that TNF-α and IL-8 may be responsible for the inhibition of neutrophil migration found in septicaemia or endotoxaemia. Selective control of TNF-α and IL-8 or blockade of their effects on neutrophil migration may be clinically useful in reestablishing the cell defence mechanisms impaired in inflammatory diseases.

### References

1. Van Dijk WC, Verbrugh HA, van der Tol ME, *et al.* Interactions of phagocytic and bacterial cells in patients with bacteremia caused by Gram-negative rod. *J Inf Dis* 1980; **141(4)**: 441–448.
2. Smith MJH, Ford-Hutchinson AW, Walker JR. Anti-inflammatory activity of bacterial endotoxin. *J Pharm Pharmacol* 1977; **29**: 702–704.
3. Rocha NP, Ferreira SH. Restoration by levamisole of endotoxin inhibited neutrophil migration, oedema and increased vascular permeability induced by carrageenin. *Eur J Pharmac* 1986; **122**: 87–92.
4. Territo MC, David WG. Granulocyte function in experimental human endotoxemia. *Blood* 1976; **47(4)**: 539–544.
5. Ellis M, Gupta S, Galant S, *et al.* Impaired neutrophil function in patients with AIDS or AIDS-related complex: A comprehensive evaluation. *J Inf Dis* 1988; **158**: 1268–1276.
6. Ward PA, Berenberg JL. Defective regulation of inflammatory mediators in Hodgkin's disease. *N Engl J Med* 1974; **290(2)**: 76–80.
7. Pereira MAA, Sannomiya P, Leme JG. Inhibition of leukocyte chemotaxis by factor in alloxan-induced diabetic rat plasma. *Diabetes* 1987; **36**: 1307–1314.
8. Ward PA, Goralnick S, Bullock WE. Defective leukotaxis in patients with lepromatous leprosy. *J Lab Clin Med* 1976; **87**: 1025–1032.
9. Donabedian H. Human mononuclear cells exposed to staphylococci rapidly produce an inhibitor of neutrophil chemotaxis. *J Inf Dis* 1985; **151(1)**: 24–32.
10. Goetzl EJ, Austen KF. A neutrophil-immbolizing factor derived from human leukocytes. *J Exp Med* 1972; **136**: 1564–1580.
11. Cunha FQ, Souza GEP, Souza CAM, Cerqueira BCS, Ferreira SH. In-vivo blockage of neutrophil migraion by LPS is mimicked by a factor released from LPS-stimulated macrophages. *Br J Exp Path* 1988; **70**: 1–8.
12. Ming WJ, Bersani L, Mantovani A. Tumour necrosis factor is chemotactic for monocytes and polymorphonuclear leukocytes. *J Immunol* 1987; **138**: 1469–1474.
13. Figari IS, Mori N, Palladino M. Regulation of neutrophil migration and superoxide production by recombinant tumor necrosis factor-α and β: Comparison to recombinant interferon-γ and interleukin-1α. *Blood* 1987; **70(4)**: 979–984.
14. Yoshimura T, Matsushima K, Tanaka S, *et al.* Purification of a human monocyte-derived neutrophil chemotactic factor that has peptide sequence similarity to other host defense cytokines. *Proc Natl Acad Sci USA* 1987; **84**: 9233–9237.
15. Nathan CF. Neutrophil activation on biological surfaces. Massive secretion of hydrogen peroxide in response to products of macrophages and lymphocytes. *J Clin Invest* 1987; **80**: 1550–1560.
16. Colditz I, Zwahlen R, Dewald B, Baggiolini M. *In vivo* inflammatory activity of neutrophil-activating factor, a novel chemotatic peptide derived from human monocytes. *Am J Pathol* 1989; **134**: 755–761.
17. Larrick JW, Graham D, Toy K, Lin LS, Senyk G, Fendly BM. Recombinant tumor necrosis factor causes activation of human granulocytes. *Blood* 1987; **69**: 640–644.
18. Lindley I, Aschauer H, Seifert JM, *et al.* Synthesis and expression in *Escherichia coli* of the gene encoding monocyte-derived neutrophil-activating factor: Biological equivalence between natural and recombinant neutrophil activating factor. *Proc Natl Acad Sci USA* 1988; **85:** 9199–9203.
19. Shalaby MR, Aggarwall BB, Rinderknecht E, Syedersky LP, Finkle BS, Palladino MA. Activation of human polymorphonuclear neutrophil functions by interferon-gamma and tumor necrosis factors. *J Immunol* 1985; **135:** 2069–2073.
20. Salyer JL, Bohnsack JF, Knape WA, Shigeoka AO, Ashwood ER, Hill HR. Mechanisms of tumor necrosis factor-α alteration of PMN adhesion and migration. *Am J Pathol* 1990; **136:** 831–841.
21. Atkinson YH, Marasco WA, Lopes AF, Vadas MA. Recombinant human tumor necrosis factor-α. Regulation of N-formylmethionylleucylphenylalanine receptor affinity and function on human neutrophils. *J Clin Invest* 1988; **81:** 759–765.
22. Kownatzki E, Kapp A, Uhrich S. Modulation of human neutrophilic granulocyte functions by recombinant human tumor necrosis factor and recombinant human lymphotoxin. Promotion of adherence, inhibition of chemotactic migration and superoxide anion release from adherent cells. *Clin Exp Immunol* 1988; **74:** 143–148.
23. Gimbrone Jr. MA, Osbin MS, Brock AF, *et al.* Endothelial interleukin-8: A novel inhibitor of leukocyte-endothelial interactions. *Science* 1989; **246:** 1601–1603.
24. Otsuka Y, Nagano K, Hori K, *et al.* Inhibition of neutrophil migration by tumor necrosis factor. *Ex vivo* and *in vivo* studies in comparison with *in vitro* effect. *J Immunol* 1990; **145:** 2639–2643.
25. Hechtman DH, Cybulski MI, Fuchs HJ, Baker JB, Gimbrone MA. Intravascular IL-8. Inhibitor of polymorphonuclear leukocyte accumulation at sites of acute inflammation. *J Immunol* 1991; **147:** 883–892.
26. Shalaby MR, Palladino Jr. MA, Hirabayashi SE, *et al.* Receptor binding and activation of polymorphonuclear neutrophils by tumor necrosis factor alpha. *J Leuk Biol* 1987; **41:** 196–204.
27. Fast DJ, Schlievert PM, Nelson RD. Nonpurulent response to toxic shock syndrome toxin 1-producing *Staphylococcus aureus*. Relationship to toxin-stimulated production of tumor necrosis factor alpha. *J Immunol* 1988; **140:** 949–953.
28. Ferrante A, Nondooskar M, Walz A, Goh DHB, Kowanko IC. Effects of tumor necrosis factor alpha and interleukin-1 alpha and beta on human neutrophil migration, respiratory burst and degranulation. *Int Arch Allergy Appl Immunol* 1988; **86:** 82–91.
29. Newman I, Wilkinson PC. Chemotactic activity of limphotoxin-a and tumor necrosis factor alpha for human neutrophils. *Immunology* 1989; **66:** 318–320.
30. Wilkinson PC, Allan RB. Assay systems for measuring leukocyte locomotion: an overview. In: Gallin JI, Quie PG, eds. *Leukocyte Chemotaxis*. New York: Raven Press, 1987; 1–23.
31. Ulich TR, del Catillo J, Ni RX, Bikhazi N. Hematologic interactions of endotoxin, tumor necrosis factor alpha (TNF alpha), interleukin 1, and adrenal hormones and hematologic effects of TNF alpha in *Coryneacterium parvum*-primed rats. *J Leukoc Biol* 1989; **45(6):** 546–557.
32. Jagles MA, Hugli TE. Neutrophil chemotactic factors promote leukocytosis. A common mechanism for recruitment from bone marrow. *J Immunol* 1992; **148(4):** 1119–1128.
33. Van Zee KJ, Fischer E, Hawes AS, *et al.* Effects of intravenous IL-8 administration in nonhuman primates. *J Immunol* 1992; **148(6):** 1746–1752.
34. Smith CW, Kishimoto TK, Abbass O, *et al.* Chemotactic factors regulate lectin adhesion molecule I (LECAM-1)-dependent neutrophil adhesion to cytokine-stimulated endothelial cells *in vitro*. *J Clin Invest* 1991; **87:** 609–618.
35. Rot A. Chemotactic potency of recombinant human neutrophil attractant/activation protein-1 (interleukin-8) for polymorphonuclear leukocytes of different species. *Cytokine* 1991; **3:** 21–26.
36. Wallis WJ, Hickstein DD, Schwarts BR, *et al.* Monoclonal antibody-defined functional epitopes on adhesion-promoting glycoprotein complex (CDw18) of human neutrophils. *Blood* 1986; **67:** 1007–1013.
37. Beutler B, Cerami A. Cachectin and tumour necrosis factor as two sides of the same biological coin. *Nature* 1986; **320:** 584–588.
38. Tracey KJ, Lowry SJ, Cerami A. Cachectin: A hormone that triggers acute shock and chronic cachexia. *J Infect Dis* 1988; **157:** 413–420.
39. Tracey KJ, Fong Y, Hesse GD, *et al.* Anti-cachectin/TNF monoclonal antibodies prevent septic shock during lethal bacteraemia. *Nature* 1987; **330:** 662–664.
40. Van Zee KJ, DeForge LE, Fischer E, *et al.* IL-8 in septic shock, endotoxemia, and after IL-1 administration. *J Immunol* 1991; **146(10):** 3478–3482.

ACKNOWLEDGEMENTS. We thank Dr Michael A. Palladino Jr. for permitting us to use his laboratories at Genentech Inc. in which these studies were carried out. We also wish to thank Dr Palladino for his valuable criticism and discussion in the preparation of this manuscript. Wirla M. S. C. Tamashiro thanks FAPESP and CNPq for supporting her study.

**Received 20 August 1992; accepted in revised form 23 September 1992**
